# Genotypic and neuropathological characterization of *Toxoplasma gondii* in striped dolphins (*Stenella coeruleoalba*) stranded along the coasts of Southern Italy

**DOI:** 10.3389/fvets.2026.1798649

**Published:** 2026-07-08

**Authors:** Emanuele Esposito, Camilla Testori, Virginia Mattioda, Doriana Iaccarino, Maria Oliviero, Gianluigi Paduano, Clementina Auriemma, Amalia Gallo, Maria Gabriella Lucibelli, Marita Georgia Riccardi, Giorgia Borriello, Giorgio Galiero, Orlando Paciello, Federica Giorda, Giuseppe Lucifora, Fabio Di Nocera

**Affiliations:** 1 Istituto Zooprofilattico Sperimentale del Mezzogiorno, Portici, Italy; 2 Istituto Zooprofilattico Sperimentale del Piemonte, Liguria e Valle d’Aosta, Torino, Italy

**Keywords:** cetaceans, genotype, neuropathology, striped dolphin, *Toxoplasma gondii*

## Abstract

*Toxoplasma gondii* is an emerging cause of neurologic disease in cetaceans. In the present study, we combined molecular detection, microsatellite genotyping, and neuropathology in stranded striped dolphins along the coasts of Campania and Calabria (Southern Italy). During the years 2018–2023, 93 cetaceans were necropsied. Brain tissues were screened for *T. gondii* DNA by Real-Time PCR targeting the 529-bp repeat element; positive samples (Ct range 21–30) were genotyped using eight microsatellite markers (TUB2, W35, TgM-A, B18, B17, M33, IV.1 and XI.1) and compared with reference strains by hierarchical clustering. Histology and immunohistochemistry (IHC) for *T. gondii* were performed on available brains. *T. gondii* DNA in the brain was detected in 12/93 cetaceans; all positive cases were striped dolphins (*Stenella coeruleoalba*). Genotyping revealed two genotypes, with GII in 10/12 dolphins and GIII in 2/12. In 10 evaluable cases, lesions ranged from mild non-suppurative meningitis/meningoencephalitis to severe necrotizing encephalitis. IHC was positive in 7/10 positive cases; tissue cysts were observed in proximity to lesions in the more severely affected brains. These findings document the circulation of multiple *T. gondii* genotypes in pelagic dolphins from Southern Italy, supporting the land-to-sea transmission hypothesis and confirming the value of striped dolphins as sentinels of terrestrial pathogens from a One Health perspective.

## 1 Introduction

*Toxoplasma gondii* is a worldwide coccidian parasite belonging to the phylum Apicomplexa with domestic and wild felids as a definitive hosts and a wide range of warm-blooded animals, including humans, as the intermediate hosts (1–4). Toxoplasmosis is a globally distributed disease occurring in terrestrial, aquatic, and marine ecosystems. In marine environments, contamination is mainly attributed to the “land-to-sea transfer” of *T. gondii* oocysts (5–8) through untreated wastewater, river runoff, and drainage systems (9–12). This spread can also involve other zoonotic pathogens, such as fecal bacteria, for example *Salmonella* spp., *Escherichia coli*, *Vibrio cholerae*, including neurotropic pathogens such as *Brucella* and *Listeria monocytogenes*, and viral agents causing hepatitis A. Being predators at the top of the marine food chain, cetaceans are more exposed to terrestrial pathogens and environmental contaminants of human origin, which are persistent, immunotoxic, neurotoxic, and endocrine disruptors, accumulating in their tissues and causing infection and often subsequent death (13). Although the transmission routes are well known in terrestrial animals (14), for marine mammals the situation is still unclear. During the last two decades, increasing reports of infection in cetaceans have raised concerns for both species conservation and public health (15, 16).

While *T. gondii* has been widely reported in an extended range of different odontocete and mysticete species in the Mediterranean Sea (17–21), however, important questions remain regarding infection pathways in offshore species (12). While the “land-to-sea transfer” hypothesis may explain infection in coastal cetaceans, such as bottlenose dolphins, it does not fully clarify how offshore species, including striped dolphins (*Stenella coeruleoalba*), acquire the parasite (17, 22). Therefore, an “alternate” *T. gondii* marine lifecycle involving pelagic cetaceans should not be ruled out (19, 23) and difference in genes coding for virulence factors should be investigated between coastal and offshore species (17, 19, 23). Concerning the pathological aspect, frequently this protozoan may act as a primary neurotropic agent causing severe, multifocal, non-suppurative meningoencephalitis (17, 19, 24, 25). In literature, the main lesions associated with toxoplasmosis in cetaceans brain are non-suppurative meningoencephalitis, perivascular cuffs of mononuclear origin, necrosis and gliosis; in some cases, intra- or extracellular protozoan cysts can be observed (12). From a genetic point of view, over 180 genotypes of *T. gondii* have been identified worldwide based on allelic variations, enhancing our understanding of the infection’s origin and its trophic transmission pathway (26). Several genotypes of *T. gondii* were described in literature; in addition to the classical types (GI, GII and GIII), atypical and African mixed/recombinant genotypes were also identified (27). GI strains are considered highly virulent and pathogenic, while GII strains are the most widespread worldwide, especially in Europe, where they cause asymptomatic cases but are considered pathogenic for immature fetuses and immunocompromised individuals. Finally, GIII strains are considered avirulent (28–30). Considering cetaceans in the Mediterranean Sea, all three genotypes and different atypical strains have been described so far (8, 19, 25, 31). Nonetheless, information regarding the genetic variability of *T. gondii* strains infecting cetaceans is limited; while certain genotypes are more common in specific regions, dolphins cover vast areas, and their migratory patterns could play a significant role in shaping the parasite’s potential diversity (8). Despite increasing reports of *T. gondii* infection in Mediterranean cetaceans, several aspects remain insufficiently investigated. In particular, data from Southern Italy are still limited, and few studies have combined molecular characterization with detailed neuropathological evaluation in the same individuals. Furthermore the distribution of circulating allelic profiles and their potential association with lesion patterns in offshore species such as the striped dolphin are not yet fully understood.

A clearer characterization of parasite genetic variability at the regional level, together with a systematic assessment of central nervous system lesions, may contribute to improving our understanding of the epidemiology and pathological significance of toxoplasmosis in pelagic cetaceans. Based on previous evidence of *T. gondii* neurotropism in marine mammals, we hypothesized that infection in stranded striped dolphins would be associated with inflammatory and necrotizing lesions consistent with protozoarian meningoencephalitis.

Therefore, the aim of the present study was to: determine the occurrence of *T. gondii* DNA in the brains of stranded cetaceans along the coasts of Campania and Calabria (Southern Italy) between 2018 and 2023, characterize the allelic profiles of positive samples using a panel of microsatellite markers in order to assess genotype-level, and correlate molecular findings with histopathological and immunohistochemical features of the central nervous system.

## 2 Materials and methods

### 2.1 Post-mortem examination

According to D.D.G.R.C. 231/2015 (32), L.R. 7/2023 (33) and DCA 130/2016 (34) on a total of 129 cetaceans stranded dead along the coasts of Campania and Calabria between 2018 and 2023, a complete post-mortem examination, according to internationally standardized guidelines (35, 36), was conducted on 93 individuals by the Istituto Zooprofilattico Sperimentale del Mezzogiorno (IZSM) in collaboration with the Italian National Reference Centre for Diagnosis of stranded Marine Mammals (C.Re.Di.Ma). Unfortunately, the remaining 36 untreated specimens were in an advanced state of decomposition and therefore unsuitable for a complete investigation to determine the cause of death.

General data such as species identification, sex, total body length (TBL) (cm), weight (kg), age class (35, 37–40), decomposition condition category (DCC) (35, 36), nutritional condition category (NCC) (36), and gastric contents were collected during necropsy.

Depending on TBL, and considering the maturation of the gonads, it was possible to establish three estimated age classes: newborn/calf, juvenile, and adult (35, 36, 38–40). Anatomical indicators, such as the blubber thickness, the rib prominence, and the convexity of the back muscle mass, allowed the evaluation of the NCC and the subsequent classification as good, moderate, and poor (37). The gastric chambers were opened to evaluate the contents such as food, foreign bodies, and the presence of parasites. During the necropsy, sampling was adapted based on DCC. For animals classified as extremely fresh or just dead (code 1), fresh (code 2), and with moderate decomposition (code 3), the tissue samples of all major organs were collected and split for various investigations, such as microbiological, biomolecular, and histopathological. Concerning biomolecular investigations, aliquots were kept frozen at – 80 °C and subsequently analyzed to assess the presence of pathogens of major concern in cetaceans including *T. gondii*. In detail, brain samples taken from all the study cases (*n* = 93) were systematically analyzed to assess the presence of the aforementioned protozoan.

When possible, the Central Nervous System (CNS) was sampled and preserved in 10% buffered formalin for subsequent histopathological and immunohistochemical (IHC) analyses carried out by C. Re. Di. Ma. At the time of recovery, each case was assigned both an IZS identification code and the Banca Dati Spiaggiamenti (BDS) code,^1^ subsequently all cases were coded in chronological order for this study (Table 1).

**Table 1:** Detailed stranding information of 12 striped dolphins tested positive for *T. gondii*.

Case ID	IZSM code	BDS n°	Stranding date	Stranding location	Region	Sex	Age class	DCC	NCC	References
1	75734	12799	26/06/2018	Bivona (VV)	Calabria	Male	Adult	2	Poor	(8, 47)
2	5248	12882	09/01/2019	Marina di Nicotera (VV)	Calabria	Female	Juvenile	1	Poor	/
3	16153	12895	29/01/2019	Palmi (RC)	Calabria	Male	Juvenile	2	Moderate	/
4	44648	13106	03/04/2019	Mondragone (Ce)	Campania	Female	Adult	3	Good	(81)
5	67143	12978	09/06/2019	Brancaleone (RC)	Calabria	Female	Adult	2	Good	/
6	115802	13097	26/10/2019	Belvedere Marittimo (CS)	Calabria	Male	Adult	2	Poor	(8)
7	27067	13158	26/02/2020	Villa San Giovanni (RC)	Calabria	Female	Juvenile	3	Good	(8)
8	178155	13316	10/12/2020	Ispani (Sa)	Campania	Male	Adult	4	Poor	(8)
9	19918	13360	11/02/2021	Bacoli (Na)	Campania	Male	Adult	2	Good	(8, 44, 81)
10	33227	13730	11/03/2023	Scalea (CZ)	Calabria	Male	Juvenile	2	Poor	/
11	33228	13731	11/03/2023	Pizzo Calabro (VV)	Calabria	Male	Adult	4	Moderate	/
12	33229	13737	19/03/2023	Lamezia Terme/Maida (CZ)	Calabria	Female	Juvenile	4	Moderate	/

### 2.2 DNA extraction and real-time PCR

Brain samples were collected during necropsies, and aliquots were frozen at −20 °C in individual tubes for genomic DNA extraction. All CNS samples were screened for the most common neurotropic pathogens for cetaceans, including *T. gondii*, *Herpesvirus* (HV), *Cetacean Morbillivirus* (CeMV), and *Brucella* spp. (BRC) (41–46).

Twenty-five mg of each tissue was homogenized with Tissue Lyser (Qiagen, Hilden, Germany), in sterile PBS buffer and with a glass bead (5 mm). Two hundred microliter of homogenate was used for nucleic acid extraction by QIAsymphony SP/AS automated instrument with a commercial kit QIAsymphomy DSP Virus/Pathogen mini kit (Qiagen, Hilden, Germany) according to the manufacturer’s instructions. *T. gondii* RH genomic DNA obtained from the America type Culture Collection (ATCC 50174D LGC Standards Italy), sent as dried microbial DNA, was used as reference material.

*T. gondii* detection was carried out with a modified Real-Time PCR protocol targeting a 529-bp repeat element (43). Compared to the original protocol by Edvinsson et al. (43), modifications were made to the thermal profile to optimize amplification conditions, and a non-competitive internal amplification control (nIAC) was implemented to monitor for PCR inhibition. Reactions were performed on a Quantstudio 1 (Thermo Fisher Scientific, Waltham, Massachusetts, United States) in a total volume of 20 μL containing 2 μL of DNA extracted, 4 μL of H_2_O, 0.5 μL of each primer at a concentration of 20 μM, 0.5 μL of Taqman probe at a concentration of 10 μM (5′–FAM CTCTCCTCCAAGACGGCTGG-BHQ-3′), 2 μL of 10 X Exo IPC Mix, 0.5 μL of 50X Exo IPC DNA and 10 μL of TaqMan Universal PCR Master Mix (Thermo Fisher Scientific, Waltham, Massachusetts, United States). Primers were forward AF1 (5′-CACAGAAGGGA CAGAA GT-3′) and reverse AF2 (5′-TCGCCTTCATCTACAGTC-3′). All reactions were run in duplicate. An internal amplification control (TaqMan exogenous IPC reagents; Thermo Fisher Scientific), detected with a VIC fluorochrome, was used in Taqman assays to investigate the presence of inhibitors in the tested sample. Amplification conditions had initial Taq DNA Polymerase activation at 50 °C for 2 min, initial denaturation at 95 °C for 10 min, 40 PCR cycles of 95 °C for 15 s and 60° for 1 min.

### 2.3 Histological and immunohistochemical findings

Based on the DCC of the carcasses, brain samples were collected during necropsies and fixed in 10% neutral buffered formalin for subsequent histological and immunohistochemical (IHC) analysis that were carried out by C. Re. Di. Ma. When available, ten areas of the central nervous system (CNS) were sampled and examined: basal nuclei, thalamus, mesencephalon, pons, obex, spinal cord, and frontal, parietal, occipital, and cerebellar cortex. After been fixed in 10% neutral buffered formalin, tissue samples were embedded in paraffin, cut to 4 ± 2 μm thick, stained with hematoxylin and eosin (H&E), and examined under a light microscope (47). IHC for *T. gondii* was conducted only on PCR-positive brain tissues or when histological changes suggestive of the infection were present using, until 2021, a polyclonal serum of caprine origin (1:500; VMRD Inc., Pullman, WA); subsequently, due to its absence on the market, samples were analyzed by means of a rabbit anti-*T. gondii* polyclonal antibody (1:500; Abcam ab138698). Morbillivirus IHC was also carried out on all samples by means of a monoclonal anti-canine distemper virus (CDV) antibody (VMRD, Pullman, WA, United States).

### 2.4 Genotyping analysis of *Toxoplasma gondii*

Analysis for the genetic characterization of *T. gondii* was performed exclusively on samples that provided a complete genetic profile. Specifically, only samples with a Ct value < 33 in Real-Time PCR were selected for genotyping, as this threshold was found to be necessary to ensure reliable and high-quality results in subsequent microsatellite fragment analysis. Due to internal protocols, before genotyping analysis, the samples were recorded with another identification code (nr. 43,999). Microsatellite genotyping by multiplex PCR and fragment analysis (TUB2, W35, TgM-A, B18, B17, M33, IV.1 and XI.1) was carried out (48). PCR was performed in a 25 μL reaction mixture consisting of 12.5 μL of 2X Qiagen Multiplex PCR Master Mix (Qiagen, Hilden, Germany), 5 μL of Q solution 1X and 0.04 μM of each primer. Five microliters of DNA from positive samples were added to the reaction mixture. DNA from ATCC strains, PTG type II (ATCC: 50941), CTG type III (ATCC: 50842) and atypical strain MAS (ATCC: 50870), already genotyped previously (49) were used as positive controls. In each PCR reaction a negative control was added, represented by RNase/DNase free water (NC). The amplification was carried out in a SimpliAmp Touch (ThermoFisher Scientific, Waltham, MA, United States) thermal cycler and consisted of an initial denaturation at 95 °C for 15 min followed by 45 cycles consisting of 94 °C for 30 s, 55 °C for 3 min and 72 °C for 60 s. A final extension step was carried out at 60 °C for 30 min. One microliter of PCR product was mixed with 0.3 μL of LIZ 500 Standard Size and 13.7 μL of HIDi Formamide. The mixture was then denatured for 5 min at 95 °C and analyzed by capillary electrophoresis with an ABI PRISM 3500 genetic analyzer (ThermoFisher Scientific, Waltham, MA, United States) equipped with a 50 cm long capillary filled with POP-7 separation medium. The GeneMapper v5.0 software was used for microsatellite fragment analysis. The minimum fluorescence threshold for valid peaks was set at 200 RFU. The hierarchical clustering analysis based on microsatellite allele profiles was performed using Ward’s method, according to (50), to assess the MS variability of the *Toxoplasma* strains detected. Genotypes belonging to reference strains present in the literature were also included in the analysis (49).

## 3 Results

### 3.1 Post-mortem examination

During the study period, based on logistical availability and according to the DCC of the carcasses, it was possible to perform a complete post-mortem examination on 93 cetaceans out of a total of 129 stranded dead along the coasts of Campania and Calabria. Of them, the majority were striped dolphins (*Stenella coeruleoalba*; *n* = 70); followed by common bottlenose dolphins (*Tursiops truncatus*; *n* = 12); sperm whales (*Physeter macrocephalus*; *n* = 4); fin whales (*Balaenoptera physalus*; *n* = 3); long-finned pilot whale (*Globicephala melas*; *n* = 1); false killer whale (*Pseudorca crassidens*; *n* = 1) and Risso’s dolphin (*Grampus griseus*; *n* = 2) (Table 2).

**Table 2:** Cetaceans species recovered along the coasts of Campania and Calabria and examined by the IZSM between 2018 and 2023.

Species	2018	2019	2020	2021	2022	2023	Total species for year
*S. coeruleoalba*	12	16	18	11	2	11	70
*T. truncatus*	2	4	0	3	1	2	12
*P. macrocephalus*	2	1	0	1	0	0	4
*B. physalus*	0	1	1	1	0	0	3
*G. melas*	0	1	0	0	0	0	1
*P. crassidens*	0	1	0	0	0	0	1
*G. griseus*	0	0	1	1	0	0	2
Total stranding for year	16	24	20	17	3	13	93

According to internationally standardized guidelines (36), during necropsy examinations, tissue samples from all main organs were collected into aliquots for subsequent routine analyses. The number and type of analyses were mainly influenced by the DCC of the carcasses at the time of necropsy. The results of biomolecular investigation for *T. gondii* in the CNS highlighted, respectively, 9 positive cases stranded along the coasts of Calabria and 3 positive cases along the coasts of Campania, for a total of 12 positive cases (12/93) identified during the study period. All positive cases were striped dolphins (Figure 1).

**Figure 1:**
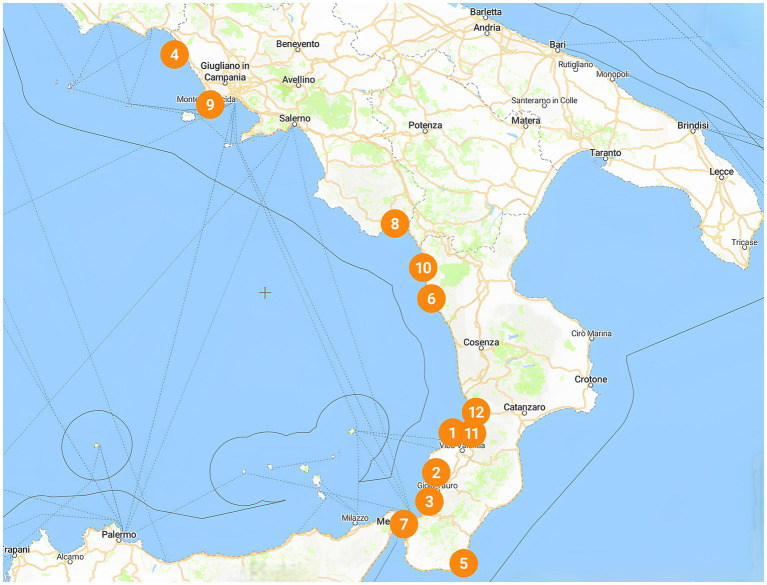
Geographical distribution of the stranding sites of the 12 striped dolphins that tested positive for *T. gondii* during the study period.

Among the study cases, 58.3% (7/12) were males and 41.7% (5/12) were female. Considering the estimated age class, 58.3% (7/12) of cases were classified as adults and 41.7% (5/12) as juvenile. The DCC of the carcasses at the time of necropsy was classified as DCC 1 in 8.3% (1/12) of cases, as DCC 2 in half of cases 50.0% (6/12), DCC 3 in 16.7% (2/12) of cases and as DCC 4 in 25.0% (3/12). Instead, the NCC of the specimens was classified as Poor in 41.7% (5/12), as Moderate in a few cases 25.0% (3/12) and as Good in 33.3% (4/12).

More detailed information about the stranding and the specimens tested positive for *T. gondii* are reported in Table 1.

As regards the coinfections, 4/12 (33.3%) cases were positive to other pathogens, as shown in Table 3. In particular, 2/12 cases presented a coinfection only with CeMV, 1/12 a positivity to both CeMV and γ-HV, and 1/12 cases was positive to BRC and α-HV.

**Table 3:** *T. gondii* associated lesions, presence of tissue cysts of protozoan origin and IHC positivity in the brain of the study cases.

Case ID	Morphological diagnosis	*T. gondii*-like cysts observed in the brain	IHC positivity for *T. gondii*	Coinfections
1	Focal mild non suppurative meningoencephalitis	No	−	γ HV, CeMV
2	Diffuse moderate non suppurative meningoencephalitis	No	−	/
3	Multifocal mild non suppurative meningoencephalitis	Yes	+	/
4	Diffuse moderate non suppurative meningoencephalitis	No	−	/
5	Diffuse severe non suppurative encephalitis associated with mild meningitis	Yes	+	/
6	Diffuse severe multifocal necrotizing encephalitis associated with mild meningitis	Yes	+	/
7	Diffuse moderate non suppurative meningoencephalitis associated with mild choroiditis	Yes	+	CeMV
8	Severe necrotizing encephalitis associated with mild non suppurative meningitis	Yes	+	CeMV
9	Diffuse severe non suppurative meningoencephalitis associated with non suppurative choroiditis.	Yes	+	α HV, BRC
10	Diffuse mild non suppurative encephalitis	No	+	/
11	Not assessed	Not assessed	Not assessed	/
12	Not assessed	Not assessed	Not assessed	/

### 3.2 Detection and genotyping analysis of *Toxoplasma gondii*

The 12 striped dolphins brains that tested positive for *T. gondii* in Real-Time PCR showed Ct values relative to the 529 bp element target (Table 4). The Ct value obtained for internal control in all samples averaged 26.41, indicating that the samples were not inhibited.

**Table 4:** Genotyping results of 12 analyzed strains (ID 43999) and 24 reference strains with 8 STRs markers.

ID_Sample	TUB2	W35	TgM-A	B18	B17	M33	IV.1	XI.1	Genotype
43999_1	289	242	207	158	336	169	274	356	GII
43999_4	289	242	207	158	336	169	274	356	GII
43999_5	289	242	207	158	336	169	274	356	GII
43999_2	289	242	207	158	336	169	274	356	GII
43999_6	289	242	205	160	336	165	278	356	GIII
43999_7	289	242	207	158	336	169	274	356	GII
43999_8	289	242	207	158	336	169	274	356	GII
43999_3	289	242	207	158	336	169	274	356	GII
43999_9	289	242	207	158	336	169	274	356	GII
43999_11	289	242	207	158	336	169	274	356	GII
43999_12	289	242	205	160	336	165	278	356	GIII
43999_10	289	242	207	158	336	169	274	356	GII
G II – PTG	289	242	207	158	336	169	274	356	GII
G III – CTG	289	242	205	160	336	165	278	356	GIII
G II – NTE	289	242	207	158	336	169	274	356	GII
G III – NED	289	242	205	160	336	165	274	356	GIII
Atypical – CASTELLS	287	242	207	158	358	169	274	356	Atypical
G I – CT1	291	248	209	160	342	169	274	358	GI
G I – GIL	291	248	209	160	342	169	274	358	GI
G III – M7741	289	242	205	160	336	165	278	356	GIII
Atypical – TgCatBr1	289	242	205	160	342	165	278	356	Atypical
G II – BOU	289	242	207	158	336	169	274	356	GII
G I – BK	291	248	209	160	342	169	274	356	GI
G III – VEG	289	242	205	160	336	165	278	356	GIII
Atypical – GUY-COE	289	246	203	160	337	165	274	356	Atypical
Atypical – GUY-MAT	291	242	203	160	339	165	272	358	Atypical
Atypical – RUB	289	242	205	160	360	167	274	356	Atypical
Atypical – GPHT	291	248	205	160	342	165	274	354	Atypical
Atypical – BOF	291	248	205	160	342	165	274	354	Atypical
Atypical – CAST	291	242	205	158	342	167	276	356	Atypical
Atypical – TgCatBr5	291	242	205	160	362	165	278	356	Atypical
Atypical – P89	291	242	205	160	348	165	278	356	Atypical
Atypical – TGCatBr3	289	242	205	160	348	165	278	356	Atypical
Atypical – VAND	291	242	203	162	344	167	276	356	Atypical
Atypical – GUY-DOS	289	246	203	160	344	167	272	356	Atypical
Atypical – COUGAR	289	242	205	158	336	169	274	354	Atypical

The analysis of STRs fragments on 8 microsatellite (MS) loci (TUB2, W35, TgM-A, B18, B17, M33, IV.1 and XI.1) was carried out on 12 samples positive for *T. gondii* by Real-Time PCR. Genotyping analysis revealed the presence of 2 distinct genotypes. Ten samples showed the same genotype belonging to type II while two samples showed a genotype corresponding to type III (Table 4). The negative control (NC) did not produce amplification peaks. Hierarchical clustering analysis confirmed the MLVA characterization of the strains under study (Figure 2), showing that all the GII isolated strains (10 out of 12) clustered together in a single, highly compact cluster, also including the GII reference strains GII_PTG, GII_NTE and GII_BOU, while the other two isolates (43999_6 and 43999_12) fell into a second cluster, including the GIII reference strains GIII_CTG, GIII_NED, GIII_M7741 and GIII_VEG.

**Figure 2:**
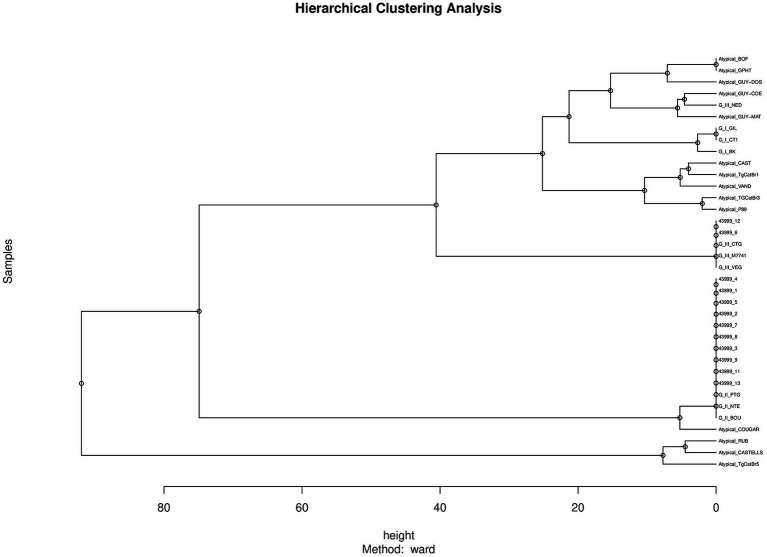
Hierarchical clustering dendrogram of microsatellite allele profiles on genotypes of 12 field strains identified in this study.

### 3.3 Histological and immunohistochemical findings

Histological and immunohistochemical examinations were performed in nearly all cases that tested positive for *T. gondii* by Real-Time PCR, with the exception of cases ID11 and ID12, where advanced carcass decomposition prevented analysis. In the remaining cases (10/12), *T. gondii* associated lesions were identified with varying degrees of severity (Table 3). In detail, 6/12 cases (ID1, ID2, ID3, ID4, ID7, and ID10) showed mild or moderate non suppurative meningoencephalitis characterized by perivascular cuffs largely composed of lymphocytes with fewer plasma cells and macrophages, and multifocal foci of gliosis. Protozoan tissue cysts were observed in cases ID3 and ID7. In case ID7, a mild non suppurative choroiditis was also observed; however, this lesion was considered more likely associated with the concurrent infection with CeMV rather than with *T. gondii*. The remaining four cases (ID5, ID6, ID8, and ID9) (Figure 3) reported more severe lesions characterized by multifocal necrosis associated with perilesional oedema, multifocal hemorrhages, neuronal necrosis and neuronophagia. In these cases, varying numbers of protozoan tissue cysts were observed, generally located in proximity to the lesions. In cases ID5, ID6 and ID8 mild non suppurative meningitis was also present. In case ID9, moderate non suppurative choroiditis was observed and was considered more likely associated with co-infection with *B. ceti* rather than *T. gondii*. Overall, the presence of tissue cysts was confirmed by IHC in 7/10 cases (Figure 3G).

**Figure 3:**
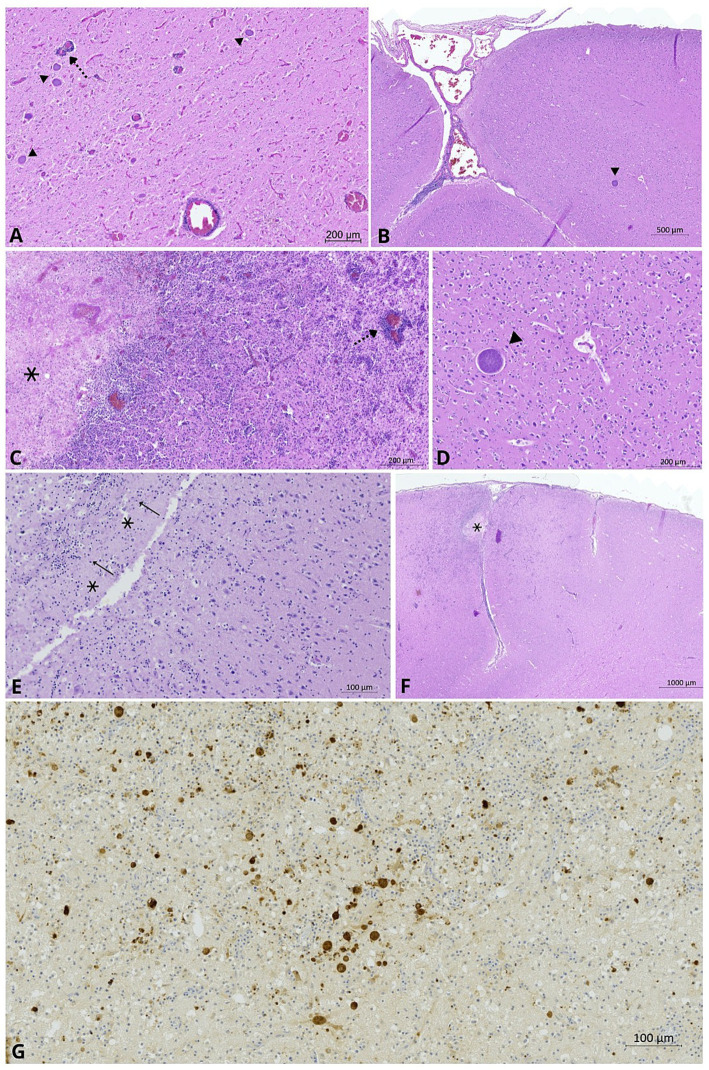
Protozoal (*T. gondii*) meningoencephalitis in stranded striped dolphins. **(A)** Multiple protozoan cysts (arrowheads) disseminated at thalamus level and focal perivascular cuffs largely composed of lymphocytes (dotted arrow) (ID9), H&E. **(B)** Non suppurative meningitis with one protozoan cyst (arrowhead) at cerebral cortex level (ID8), H&E. **(C)** Focal necrosis (asterisk) surrounded by inflammatory infiltrate mainly composed of lymphocytes, plasma cells and macrophages but also some neutrophil granulocytes especially near the necrotic center. Lymphoplasmacytic perivascular cuffs are also present in the top right at cerebral cortex level (dotted arrow) (ID6), H&E. **(D)** One protozoal cyst (arrowhead) at cerebral cortex level (ID8), H&E. **(E)** Foci of necrosis in the white matter of the cerebral cortex (asterisk) associated with lymphoplasmacytic to granulomatous inflammation; several Gitter cells are also present (arrows) (ID6), H&E. **(F)** Non suppurative meningitis associated with focal necrosis (asterisk) and pyogranulomatous inflammation of the surrounding tissue at cerebral cortex level (ID8), H&E. **(G)** Several protozoal cysts consistently immunostained for *T. gondii* antibody at thalamus level (ID6), IHC.

## 4 Discussion and conclusion

In the Mediterranean Sea, the presence of *Toxoplasma gondii* was reported in various aquatic organisms including fish (18, 51), shellfish (52–54), crab (55) and several species of cetaceans, causing subacute to chronic infections in the latter (24, 25, 56–58). Studies conducted on cetaceans stranded along the Italian and Spanish coasts indicate that the protozoan is widespread, with positivity rates ranging on average between 10.0 and 15.0% in molecular investigations, and exceeding 50.0% in serological investigations. In particular, bottlenose dolphins are the species with the highest seroprevalence (57.0%), followed by striped dolphins with serological data around 11.0–20.0% and molecular findings varying between 7.0 and 15.0% depending on the year and area of stranding (8, 59).

The results obtained in this study confirm the spread of the parasite along the Italian coastline, focusing attention on cetaceans stranded along the coasts of Campania and Calabria and providing further information on the genetic profile of the pathogen prevalent in these areas. In our study, all positive cases were identified in *S. coeruleoalba*. This finding is consistent with the high susceptibility of this species reported in the Mediterranean (24, 25, 60). Although other species were screened, the predominance of striped dolphins in our sampling (75.3%) and their specific pelagic feeding habits, potentially involving prey that concentrate oocysts, may explain why the parasite was not detected in other cetaceans with different ecological niches or smaller sample sizes. In contrast to the findings of Guardo et al. (24), the presence of the parasite in infected individuals was homogeneous, represented by a similar percentage of *T. gondii* detection between males and females and adults and juveniles as well.

Studies identifying *T. gondii* in marine animals have shown that Europe is the fourth continent with a prevalence rate of 13.0%, preceded by Antarctica (39.0%), North America (21.0%), and Oceania (18.0%), and marine mammals (cetaceans and pinnipeds) are the most affected. However, such comparisons are not feasible in the present study, as the analysis was limited to brain tissue.

The genotyping investigations of this study evidenced that most of the samples analyzed showed the GII archetype (10 out of 12), while only 2 samples showed the GIII archetype.

These results are consistent with epidemiological data observed in Europe, where clonal GII and GIII are among the most frequently reported variants in domestic animals, wildlife, and, more recently also in marine mammals (8, 61). Several microsatellite studies of *T. gondii* strains isolated from cetaceans have shown a predominance of GII and, only occasionally, GIII.

The predominance of the GII observed in this study is consistent with previous investigations conducted in Europe, where this genotype represents the most widespread variant circulating among domestic animals, wildlife, and humans. In Europe, GII is the most frequently isolated genotype in clinical cases of human congenital and ocular toxoplasmosis, as well as in the main livestock species (pigs, sheep, and poultry) intended for human consumption (62–64). In Italy, several studies have confirmed that GII, and to a lesser extent GIII, are the dominant archetypal genotype in the terrestrial environment, consistently reported in domestic ruminants and synanthropic intermediate hosts (65). GII strains have also been frequently reported in marine mammals from different geographic areas, including the Mediterranean Sea (8, 28, 61, 64).

The detection of a genotype typically associated with terrestrial transmission cycles strongly supports the hypothesis of a land-to-sea transfer of *T. gondii*. The genetic continuity between the strains identified in our marine samples and those typically associated with the Italian terrestrial “One Health” interface, including humans and domestic animals, suggests that the coastal runoff and wastewater discharge in the Southern Tyrrhenian and Ionian Seas act as a direct bridge for these specific genotypes (14, 20, 66).

Although striped dolphins (*S. coeruleoalba*) are generally considered a pelagic species with limited interaction with coastal habitats, the predominance of the GII strains in the present study suggests that terrestrial contamination may extend well beyond coastal areas and affect offshore marine ecosystems. Similar findings have been reported in other studies on marine mammals, highlighting how land-derived pathogens may disperse widely in the marine environment and infect pelagic species through complex trophic pathways (12, 26).

To date, data on the genetic characterization of *T. gondii* in cetaceans from the Southern Tyrrhenian Sea are still limited but the results presented here support the hypothesis of a terrestrial origin of the infection due to marine contamination by oocysts, as GII and, to a lesser extent, GIII (54, 56, 64).

In particular, regional studies conducted in Campania and Calabria have identified GII and GIII as the main circulating strains in wild boars (*Sus scrofa*) and various species of wild birds (67, 68). The genetic identity between these terrestrial isolates and the strains detected in striped dolphins in this study provides robust evidence for the ‘spillover’ of oocysts from the mainland to the marine ecosystems, mirroring the same parasitic population structure observed in the human, animal and environment interface of Southern Italy. The identification of GIII strains, although less frequent, highlights the circulation of multiple variants, demonstrating the importance of microsatellites studies for the epidemiological characterization of the parasite in marine ecosystems.

Concerning neuropathological findings in cetaceans, these are generally linked to four main pathogens such as CeMV, *HV*, *B. ceti* and *T. gondii* (60). In particular, most meningoencephalitis of parasitic origin in cetaceans have been associated with protozoan infections, particularly *T. gondii* (19–21, 69–71). In the present study, the analyzed cases showed different degrees of severity of neuropathological lesions associated with *T. gondii* infection, all of which were consistent with those reported in literature (12). The observed pathological spectrum ranged from mild to moderate non-suppurative inflammatory changes to severe necrotizing encephalitis, confirming the marked neurotropism of *T. gondii* in cetaceans.

The neuropathological patterns described in this study, as well as the species involved, are in line with previous reports, further supporting the well-recognized susceptibility of striped dolphins (*S. coeruleoalba*) to toxoplasmosis (8, 17, 24, 47).

Notably, case ID5, in which an allelic profile consistent with genotype III was detected, showed severe non-suppurative encephalitis associated with mild meningitis. While this represents a preliminary observation based on a single case, it suggests that infection with this genotype could potentially be associated with severe neuropathological lesions in cetaceans, despite GIII strains being generally considered less virulent in experimental murine models.

Choroiditis was observed in two animals in the presence of other neurotropic agents: CeMV in ID7 and *B. ceti* in ID9. Although cases of choroiditis associated solely with the presence of *T. gondii* have been reported in the literature (72), the lesion observed in the present cases is considered more likely to be associated with the other detected pathogens.

Due to the nature of the study, most of the animals were wild stranded dolphins found dead; the only animal found alive (ID2) did not show clinical signs referable to neurological disease at the time of the stranding. Therefore, no clinical informations prior to death were available, representing a limitation for the clinical interpretation of the findings.

The heightened susceptibility in striped dolphins (*S. coeruleoalba*) has been hypothesized to be related to the lack of a long-standing coevolutionary relationship between host and parasite, which may instead have developed in more coastal species such as the bottlenose dolphin (*T. truncatus*). Coastal species are more frequently and continuously exposed to *T. gondii* due to higher levels of environmental contamination in nearshore habitats, potentially allowing the development of a more effective immune response and better control of parasite dissemination (73).

Despite the growing body of evidence documenting toxoplasmosis in cetaceans, the transmission pathways and pathogenesis of *T. gondii* in marine mammals remain incompletely understood. At present, the most likely source of infection for cetaceans is represented by oocyst-contaminated wastewater discharged into the marine environment, including untreated effluents released from vessels (19). Indeed, it has been proven that the parasite’s oocysts can survive in seawater and remain infectious until 6 months; if, however, the seawater temperature is around 4 °C, they remain infectious for up to 24 months (74, 75).

However, additional routes of exposure cannot be excluded, and other sources of infection have been proposed, including feeding on mussels or fish contaminated with the protozoan (17). In particular, bivalve molluscs such as *Mytilus galloprovincialis*, *Tapes decussatus* and *Magallana gigas*, are capable of accumulating oocysts, thereby acting as potential vectors for the transmission of the parasite within marine aquatic animals (51, 53, 76–78).

Furthermore, several studies showed that *T. gondii* oocysts can survive for at least 8 h in the gastrointestinal tract of filter-feeding fish, such as anchovies and sardines, exposed to the parasite for at least 2 h, transforming them into vectors in the offshore marine environment (18, 79).

Finally, new hypotheses consider microplastics as transmission vehicles. It has been shown that protozoa can be associated with microplastics present in contaminated seawater and easily penetrate marine food webs. However, pathogen transport can be influenced by the movement of microplastics; if they float on the sea surface and travel long distances, they can facilitate the dispersal of pathogens to locations far from their original terrestrial source. If they sink, they can concentrate pathogens in the benthos, where many filter-feeding marine invertebrates reside, further increasing the likelihood of pathogen ingestion by these species.

In particular, microfibers, resulting from broken fishing nets, are considered the main microplastics associated with the transport of marine parasites (51, 80).

In conclusion, the findings of this study provide critical evidence of the persistence and widespread circulation of the terrestrial parasite *T. gondii* within the marine ecosystem. The identification of severe neuropathological lesions suggests that, despite the vast dilution capacity of the marine environment, even pelagic species such as the striped dolphin are subject to infectious doses sufficient to trigger clinically significant disease.

Furthermore, the detection of *T. gondii* GII and GIII in cetaceans from the Campania and Calabrian coasts highlights a direct epidemiological link with surrounding terrestrial ecosystems, reflecting the spillover of zoonotic pathogens from land to sea. This underscores the role of cetaceans as flagship sentinel species for monitoring the environmental impact of toxoplasmosis and as a proxy for evaluating public health risks.

Consequently, integrated molecular surveillance programs are essential to further elucidate the transmission dynamics, environmental drivers, and strain-specific virulence of *T. gondii* in the Mediterranean. Finally, the prevalence of terrestrial genotypes in these marine sentinels underscores the urgent need for improved wastewater management and coastal runoff mitigation strategies in the Campania and Calabria regions. Enhancing filtration systems to effectively trap *T. gondii* oocysts is a crucial step in reducing the land-to-sea pathogen flow, thereby protecting both marine biodiversity and public health within a comprehensive One Health perspective.

## Data Availability

The original contributions presented in the study are included in the article/supplementary material, further inquiries can be directed to the corresponding authors.

## Footnotes

1 http://mammiferimarini.unipv.it/, accessed on 29 December 2025.
